# Supplementary material for: Pulse Root Ideotype for Water Stress in Temperate Cropping System
Source: Plants (Basel). 2021 Apr 3;10(4):692. doi: 10.3390/plants10040692 (PMC8067327; doi:10.3390/plants10040692)
Supplement: Supplementary file 1 [file plants-10-00692-s001.zip › Supplementary/Table S1 Summary of root traits examined in studies conducted in pulses and legumes.docx]

**Supplementary Table 1**. Summary of root traits examined in studies conducted in pulses and legumes.

| **Study** | **Condition** | **DAS** | **Crop** | **RDW**  (g p^-1^) | **SDW**  (g plant^-1^) | **RLD**  (cm cm^-3^) | **TRL**  (cm) | **TpRL**  (cm) | **Diameter** (mm) | **LRN** | **RS**  (cm^-2^ p^-1^) |
| --- | --- | --- | --- | --- | --- | --- | --- | --- | --- | --- | --- |
| Beebe*, et al.* [1] | field | 35 | Bean | 2.14 - 3.22 |  | 0.36 - 0.41 | 12.58 - 40.82 |  |  |  |  |
| Beebe, Rojas-Pierce, Yan, Blair, Pedraza, Muñoz, Tohme and Lynch [1] | hydroponics | 14 | Bean | 0.14 - 0.24 |  |  | 34.48 - 68.15 | 15.81 - 19.69 |  | 2.67 - 8.33 |  |
| Benjamin and Nielsen [2] | field |  | Chickpea | 1.72 |  |  |  |  |  |  | 52.5 |
| Liu*, et al.* [3] | field |  | Chickpea |  |  | 7-8.4 |  |  | 0.38-0.49 | 2245-2727 | 11.8-10.6 |
| Varshney*, et al.* [4] |  | 35 | Chickpea | 1.10 - 1.41 |  | 0.38 - 0.44 |  |  |  |  |  |
| Chen*, et al.* [5] | nutrient solution | 28-42 | Chickpea | 1.19 - 3.70 | 0.82-10.06 |  | 305 - 3824 | 38.3 - 105 | 0.68 - 1.07, 0.87(Tp) | 23-764 | 94.8-1027 |
| Ramamoorthy*, et al.* [6] | field |  | Chickpea | ~5-34 (g m^-2^) | 6171-7928 (kg Ha^-1^) | 0.218-0.411 |  |  |  |  |  |
| Serraj*, et al.* [7] | field | 35 | Chickpea | 7.9 -14.4 | 52.1-63 | 0.162 - 0.271 |  |  |  |  |  |
| Kashiwagi*, et al.* [8] | field,  PVC cylinder vertisol and sand mix | 35 | Chickpea |  | 21.1-65.7,  43.9-118.1 | 0.15-0.58,  0.24-57 |  |  |  |  |  |
| Kashiwagi*, et al.* [9] | field | 35 | Chickpea | 0.54-0.8 | 1.09-2.10 | 0.199-0.304 |  |  |  |  |  |
| Zaman-Allah*, et al.* [10] | vertisol, potted | 94 | Chickpea | 1.93-3.03 | 9.16-11.85 |  |  | 75-120 (Rdp) |  |  |  |
| Adu [11] |  |  | Cowpea |  |  |  |  |  | 1.9-17.7 |  |  |
| Benjamin and Nielsen [2] | field | mid pod fill | Field pea | 0.67 kg m^-2^ |  |  |  |  |  |  | 13.7m^2^roots m^2^ surface area^-1^ |
| Liu, Gan, Bueckert and Van Rees [3] | field |  | Field pea |  |  | 6.7-7.9 |  |  | 0.31-0.43 | 1816-2038 | 8.6-8.7 |
| Belachew*, et al.* [12] | perlite and sand | 28 | Faba bean | 0.21 - 1.59 | 0.27-3.49 |  | ~100 - 1300 | 28.3 - 59.4, 61 - 78 (Rdp) |  |  |  |
| Futsaether and Oxaal [13] | glass beads | 20 | Lentil |  |  |  | 140 | 22 | 0.2 - 0.3,  0.5 - 0.6(Tp) |  |  |
| Gahoonia*, et al.* [14] | pot soil | 60 | Lentil |  |  |  | 17 - 24 |  |  |  |  |
| Sarker*, et al.* [15] | Pot soil:sand | 35 | Lentil | 0.12 - 0.92 |  |  | 68 - 705 | 11.60 - 47.2 |  | 16 - 50 |  |
| Gahoonia*, et al.* [16] | soil | 60 | Lentil |  |  |  | 34.94 |  |  |  |  |
| Sarker and Karmoker [17] | quartz sand |  | Lentil |  |  |  | ~12 - 20 | ~4.5 - 6.25 |  | ~6 - 10 |  |
| Liu, Gan, Bueckert and Van Rees [3] | field |  | Lentil |  |  | 5.4-10.2 |  |  | 0.42-0.38 | 1611-1960 | 7.7-13.5 |
| Idrissi*, et al.* [18] | pot experiment | 38 | Lentil | 53.46 - 124.95 |  |  |  | 21.06 - 26.32 | 0.71 - 1.05 | 34.66 -63.33 |  |
| Chen*, et al.* [19] | soil, potted | 42 | Lupin | 2.28-3.40 | 1.55-4.16 |  | 460-760 |  |  |  |  |
| Chen*, et al.* [20] |  |  | Lupin | 1.63 - 5.98 | 23 - 133 |  |  | ~10-26 | root collar 7-14, LR 0.9-1.90 |  | 125 - 339 |
| Chen*, et al.* [21] |  | 42 | Lupin | 2.49 |  |  | 416 | 26.6 | 0.97 |  | 128 |
| Benjamin and Nielsen [2] | field |  | Soybean | 5 |  |  |  |  |  |  | 13.4 |
| Manavalan*, et al.* [22] | turface:sand - cone system | 12 | Soybean | ~0.02-0.12 | ~0.04-0.2 |  |  | 9.44 - 30.18 |  |  |  |
| Liu, Gan, Bueckert and Van Rees [3] | field |  | *Wheat |  |  | 15.9-17.7 |  |  | 0.28-0.22 | 4007-4622 | 13.5-13.7 |

*for comparison. Days to harvest (DAS), root dry weight (RDW), shoot dry weight (SDW), root length density (RLD), total root length (TRL), rooting depth (Rdp) taproot (Tp), lateral root (LR), lateral root number (LRN), root surface area (RS), per plant (p^-1^).

**Reference**

1. Beebe, S.E.; Rojas-Pierce, M.; Yan, X.; Blair, M.W.; Pedraza, F.; Muñoz, F.; Tohme, J.; Lynch, J.P. Quantitative Trait Loci for Root Architecture Traits Correlated with Phosphorus Acquisition in Common Bean. *Crop Science* **2006**, *46*, 413-423, doi:10.2135/cropsci2005.0226.

2. Benjamin, J.G.; Nielsen, D.C. Water deficit effects on root distribution of soybean, field pea and chickpea. *Field Crops Research* **2006**, *97*, 248-253, doi:<https://doi.org/10.1016/j.fcr.2005.10.005>.

3. Liu, L.; Gan, Y.; Bueckert, R.; Van Rees, K. Rooting systems of oilseed and pulse crops. II: Vertical distribution patterns across the soil profile. *Field Crops Research* **2011**, *122*, 248-255, doi:<https://doi.org/10.1016/j.fcr.2011.04.003>.

4. Varshney, R.K.; Gaur, P.M.; Chamarthi, S.K.; Krishnamurthy, L.; Tripathi, S.; Kashiwagi, J.; Samineni, S.; Singh, V.K.; Thudi, M.; Jaganathan, D. Fast-Track Introgression of “QTL-hotspot” for Root Traits and Other Drought Tolerance Traits in JG 11, an Elite and Leading Variety of Chickpea. *The plant genome* **2016**, *6*, 1-9.

5. Chen, Y.; Ghanem, M.E.; Siddique, K.H. Characterising root trait variability in chickpea (Cicer arietinum L.) germplasm. *Journal of Experiental Botany* **2017**, *68*, 1987-1999, doi:10.1093/jxb/erw368.

6. Ramamoorthy, P.; Lakshmanan, K.; Upadhyaya, H.D.; Vadez, V.; Varshney, R.K. Root traits confer grain yield advantages under terminal drought in chickpea (Cicer arietinum L.). *Field Crops Research* **2017**, *201*, 146-161, doi:<https://doi.org/10.1016/j.fcr.2016.11.004>.

7. Serraj, R.; Krishnamurthy, L.; Kashiwagi, J.; Kumar, J.; Chandra, S.; Crouch, J.H. Variation in root traits of chickpea (Cicer arietinum L.) grown under terminal drought. *Field Crops Research* **2004**, *88*, 115-127, doi:<https://doi.org/10.1016/j.fcr.2003.12.001>.

8. Kashiwagi, J.; Krishnamurthy, L.; Crouch, J.H.; Serraj, R. Variability of root length density and its contributions to seed yield in chickpea (Cicer arietinum L.) under terminal drought stress. *Field Crops Research* **2006**, *95*, 171-181, doi:<https://doi.org/10.1016/j.fcr.2005.02.012>.

9. Kashiwagi, J.; Krishnamurthy, L.; Gaur, P.M.; Chandra, S.; Upadhyaya, H.D. Estimation of gene effects of the drought avoidance root characteristics in chickpea (C. arietinum L.). *Field Crops Research* **2008**, *105*, 64-69, doi:<https://doi.org/10.1016/j.fcr.2007.07.007>.

10. Zaman-Allah, M.; Jenkinson, D.M.; Vadez, V. Chickpea genotypes contrasting for seed yield under terminal drought stress in the field differ for traits related to the control of water use. *Functional Plant Biology* **2011**, *38*, 270-281, doi:<https://doi.org/10.1071/FP10244>.

11. Adu, O.M. Identifying key contributing root system traits to genetic diversity in field-grown cowpea (Vigna unguiculata L. Walp.) genotypes. *Field Crops Research* **2019**, *v. 232*, pp. 106-118-2019 v.2232, doi:10.1016/j.fcr.2018.12.015.

12. Belachew, K.Y.; Nagel, K.A.; Fiorani, F.; Stoddard, F.L. Diversity in root growth responses to moisture deficit in young faba bean (Vicia faba L.) plants. *PeerJ* **2018**, *6*, e4401-e4401, doi:10.7717/peerj.4401.

13. Futsaether, C.M.; Oxaal, U. A growth chamber for idealized studies of seedling root growth dynamics and structure. *Plant and Soil* **2002**, *246*, 221-230, doi:10.1023/a:1020609224525.

14. Gahoonia, T.S.; Ali, O.; Sarker, A.; Rahman, M.M. Root traits, nutrient uptake, multi-location grain yield and benefit–cost ratio of two lentil (Lens culinaris, Medikus.) varieties. *Plant and Soil* **2005**, *272*, 153-161, doi:10.1007/s11104-004-4573-x.

15. Sarker, A.; Erskine, W.; Singh, M. Variation in shoot and root characteristics and their association with drought tolerance in lentil landraces. *Genetic Resources and Crop Evolution* **2005**, *52*, 89-97, doi:10.1007/s10722-005-0289-x.

16. Gahoonia, T.S.; Ali, O.; Sarker, A.; Nielsen, N.E.; Rahman, M.M. Genetic Variation in Root Traits and Nutrient Acquisition of Lentil Genotypes. *Journal of Plant Nutrition* **2006**, *29*, 643-655, doi:10.1080/01904160600564378.

17. Sarker, B.C.; Karmoker, J.L. Effects of phosphorus deficiency on the root growth of lentil seedlings (Lentil culinaris Medik) grown in rhizobox. *Bangladesh Journal of Botany* **2009**, *38*, 215-218.

18. Idrissi, O.; Houasli, C.; Udupa, S.M.; De Keyser, E.; Van Damme, P.; De Riek, J. Genetic variability for root and shoot traits in a lentil (Lens culinaris Medik.) recombinant inbred line population and their association with drought tolerance. *Euphytica* **2015**, *204*, 693-709, doi:10.1007/s10681-015-1373-8.

19. Chen, Y.L.; Dunbabin, V.M.; Postma, J.A.; Diggle, A.J.; Siddique, K.H.M.; Rengel, Z. Modelling root plasticity and response of narrow-leafed lupin to heterogeneous phosphorus supply. *Plant and Soil* **2013**, *372*, 319-337, doi:10.1007/s11104-013-1741-x.

20. Chen, Y.L.; Palta, J.; Clements, J.; Buirchell, B.; Siddique, K.H.M.; Rengel, Z. Root architecture alteration of narrow-leafed lupin and wheat in response to soil compaction. *Field Crops Research* **2014**, *165*, 61-70, doi:<https://doi.org/10.1016/j.fcr.2014.04.007>.

21. Chen, Y.; Shan, F.; Nelson, M.N.; Siddique, K.H.; Rengel, Z. Root trait diversity, molecular marker diversity, and trait-marker associations in a core collection of Lupinus angustifolius. *Journal of Experimental Botany* **2016**, *67*, 3683-3697, doi:10.1093/jxb/erw127.

22. Manavalan, L.P.; Guttikonda, S.K.; Nguyen, V.T.; Shannon, J.G.; Nguyen, H.T. Evaluation of diverse soybean germplasm for root growth and architecture. *Plant and Soil* **2010**, *330*, 503-514, doi:10.1007/s11104-009-0222-8.
