# Supplementary figures and images for: Pulse Root Ideotype for Water Stress in Temperate Cropping System
Source: Plants (Basel). 2021 Apr 3;10(4):692. doi: 10.3390/plants10040692 (PMC8067327; doi:10.3390/plants10040692)

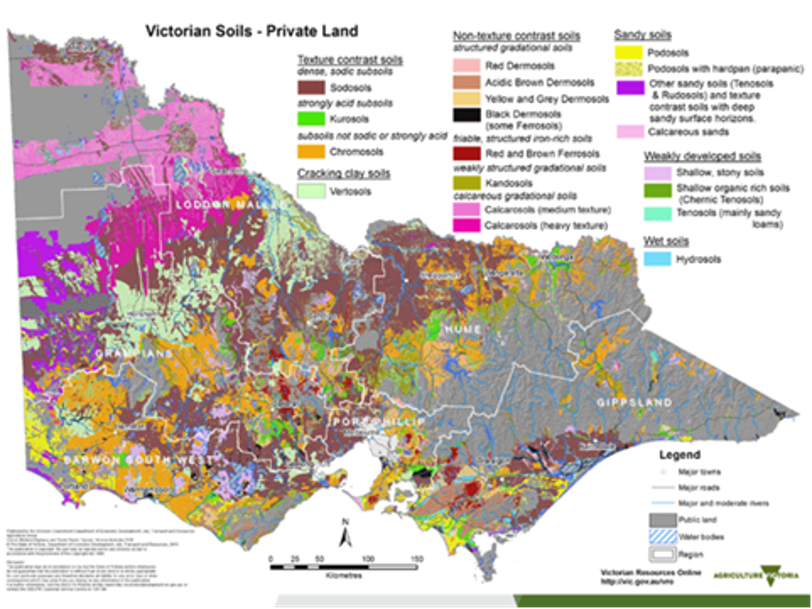

Supplement: Supplementary file 1 [file plants-10-00692-s001.zip › Supplementary/Figure S1 Victorian soils Private-land.png]

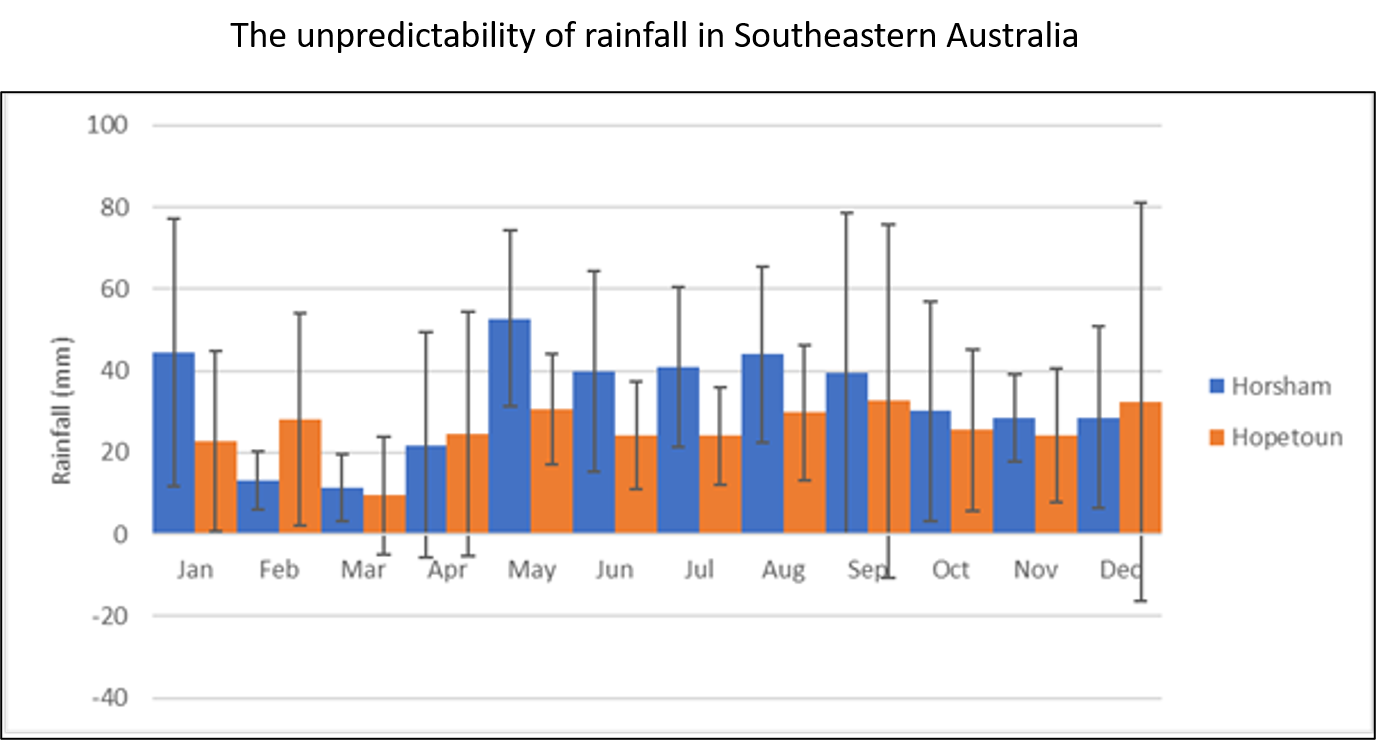

Supplement: Supplementary file 1 [file plants-10-00692-s001.zip › Supplementary/Figure S2 The unpredictability of rainfall in Southeastern Australia.png]
